# Wasting Money on Neurotics

**Published:** 1923-06

**Authors:** 


### WASTING MONEY ON THE NEUROTICS.

### WHAT A CONSULTANT THINKS.

[The writer of the following article is a consultant in an industrial region where there is loyal co-operation between the local authorities and the doctors, where the working of the National Health Insurance Acts is satisfactory, and where there is the minimum of friction. The conditions obtaining in his district are therefore not due to local circumstances, and the criticisms offered may be regarded as general in their application.]

The burden of carrying the chronic neurotic is the greatest source of waste in the working of the Insurance Act. The waste is greater than appears, because the diagnosis of neurasthenia is rarely used. Under this heading should come most of the cases of dyspepsia, gastritis, colitis, dysmenorrhœa and other gynæcological conditions, neuritis, and a host of other ailments. But the problem is of national importance, and extends far beyond that measure. We meet it in its worst form in the Workmen's Compensation Act, and it also appears in the Courts dealing with all cases of injury and compensation. It is likewise at the root of many of the grievances in the Ministry of Pensions. Altogether between the Insurance Act, with its burden on industry and on the workers, the Compensation Act, with its burden on industry, and the Pension Warrant, with its burden on the taxpayer, we cannot be far wrong in estimating neurosis as one of the great national problems.

### The War-time Neurotic.

What is a neurosis ? There is no question more difficult to answer in the whole of medicine. One or two examples will illustrate better than a technical definition. During the war men were wounded in the arm and sent home to all the comforts of hospital. It became obvious to the meanest intelligence that a "Blighty one" was a blessing in a very thin disguise. Through a psychological process now fairly well understood the unspoken wish became converted into a paralysis of the arm, and the man was sent to hospital. It was not malingering, although it was not what it was termed, "shell-shock." The instinct of self-preservation, acting unconsciously, preserved the soldier in a way which at the same time saved his honour. This process overcame some of the best of men under prolonged strain, although the large majority of cases collapsed at the first available opportunity.

### The Neurosis in Civil Life.

Similarly in civil life people who are temperamentally or physically unfit for their job in life become neurotic. Any instinct may be at the root of the neurosis, but the commonest are the instinct of self-preservation and the sexual instinct. In civil life the neurosis is much less effective for the attainment of its end than it was during the war. It is not a rational way out of the unpleasantness from which the individual suffers, because it is not due to a rational process, as malingering would be. It often fails completely. The business man who through neurosis flies from his tottering business does not save himself and the business, as would the less clever man who can look difficulties in the face. The neurotic patient who gets Sickness Benefit through a neurosis only becomes more and more unfit for his job, and tends to sink to a lower plane. The miner who, in fear of the darkness and dangers of the pit, develops a traumatic neurosis and gets compensation, is not a happier man than the man who returns to work. The symptoms are very real indeed, but they are in the nature of a delusion, which is merely strengthened by drug treatment. The patient thinks that a disease has attacked him, whereas he has sought a disease.

### What is the Remedy ?

The most potent remedy would be to refuse sick benefit, but this is impracticable at the present time. There is one difficulty which is almost insuperable when dealing with such an enormous number of cases as those which pester panel practitioners and deplete the Societies' funds. Before a neurosis can be diagnosed we must be able absolutely to exclude organic disease. In almost all organic disease the symptoms felt by the patient are the first to be noticed, and it is only by prolonged observation that neurosis can be diagnosed. The symptoms of a neurotic dyspepsia may be exactly the same as those of cancer of the stomach in an early stage. An apparently neurotic headache may turn out to be tuberculous meningitis, and a hysterical paralysis may be the early symptom of disseminate sclerosis.

### The Need for Central Surgeries.

We have all of us been deceived so many times that we hesitate to commit ourselves at an early stage. To throw the onus of refusing benefit on to a busy practitioner with a crowded waiting room is quite impossible. He requires in all cases moral support, and in many cases specialist aid in diagnosis. My suggestion would be that the best remedy lies in central consulting rooms, where the patient can be examined by two independent men, and where specialist aid would be available. Every patient on sick benefit for more than, say, three weeks would then have to receive a certificate signed by two men that the case was not one of neurosis, but of definite organic disease incapacitating the patient. From the medical point of view the scheme of central surgeries is ideal ; the average medical man likes the help of a colleague, as can be seen in hospital, where consultations are of hourly occurrence. The habit has only to be acquired to become a necessity. Once get the men to work in properly equipped rooms, and the advantages will be so obvious that the method will spread to private practice and there will eventuate the treatment centre with, perhaps, X-ray department and laboratory. Part-time specialists would soon appear upon the scene and panel practice would be revolutionised gradually. The fundamental cause of the waste exists not in the Act, but in human nature, and exactly the same waste is found outside the scope of the Act.

